# Gut bacteria promote proliferation in benign S/RG/C2 colorectal tumour cells, and promote proliferation, migration and invasion in malignant HCT116 cells

**DOI:** 10.1038/s41598-023-44130-6

**Published:** 2023-10-12

**Authors:** J. L. Robson, R. M. S. Thorn, A. C. Williams, T. J. Collard, D. Qualtrough

**Affiliations:** 1 Department of Applied Sciences, University of the West of England, Bristol, England; 2 School of Cellular and Molecular Medicine, University of Bristol, Bristol, England

**Keywords:** Cancer, Cell biology, Microbiology, Diseases, Oncology

## Abstract

Colorectal cancer (CRC) is a significant global health burden with a rising incidence worldwide. Distinct bacterial populations are associated with CRC development and progression, and it is thought that the relationship between CRC and associated gut bacteria changes during the progression from normal epithelium to benign adenoma and eventually malignant carcinoma and metastasis. This study compared the interaction of CRC-associated species Enterotoxigenic *Bacteroides fragilis*, *Enterococcus faecalis* and *Fusobacterium nucleatum* and one probiotic species, *Escherichia coli* Nissle 1917 with a colorectal adenoma (S/RG/C2) and a colorectal adenocarcinoma (HCT116) derived cell line. Gentamicin protection assays showed that all species displayed higher attachment to benign tumour monolayers when compared to malignant monolayers. However, invasion of 3/4 species was higher in the HCT116 cells than in the adenoma cells. All species were found to persist within tumour cell monolayers for a minimum of 48 h under standard aerobic cell culture conditions, with persistence significantly higher in HCT116 cells. Downstream assays were performed to analyse the behaviour of S/RG/C2 and HCT116 cells post-infection and revealed that all species increased the tumour cell yield of both cell lines. The migratory and invasive potential of HCT116 cells was increased after infection with *F. nucleatum*; however, no species significantly altered these characteristics in S/RG/C2 cells. These results add to the growing evidence for the involvement of microorganisms in CRC progression and suggest that these interactions may be dependent on tumour cell-specific characteristics.

## Introduction

Colorectal cancer (CRC) is the third highest incidence malignancy worldwide and remains a leading cause of cancer-related death^1^. CRC prevalence is predicted to rise globally, in part due to increasing economic development and the adoption of a Western diet^2^. Development of CRC normally occurs over several decades and progresses along the adenoma-carcinoma sequence as described by Fearon and Vogelstein^3^. In recent years, there has been an alarming increase in CRC incidence in younger populations^4^, highlighting the importance of environmental and lifestyle factors in colorectal carcinogenesis.

Early driver mutations, such as the inactivation of the APC destruction complex seen in > 80% of colorectal cancers, have been well defined and extensively reviewed^5–7^. However, progression from malignant tumour to metastasis is thought to be independent of mutational status and heavily influenced by the microenvironment^8^. Diet and other environmental factors have been shown to influence CRC development and progression through their effects on the composition of the gut microbiome^9,10^ although the exact mechanisms remain poorly understood^9,10^.

Numerous changes in the relative abundance of certain bacterial species, also known as microbial dysbiosis, have been described in CRC patients^11^. For example, Enterotoxigenic *Bacteroides fragilis* (ETBF) is found in 38% of CRC patients, compared to only 12% of healthy controls^12^. On this evidence, some species have emerged as CRC-associated pathogens and exhibit both direct and indirect effects on tumorigenesis. For example, colonisation by species such as ETBF sustains chronic inflammation in the colon^13^. Additionally, the production of genotoxic compounds, such as superoxide by *Enterococcus faecalis*, contributes to DNA damage and chromosomal instability^14^. Finally, ETBF and other species including *Fusobacterium nucleatum* possess virulence factors which cleave E-cadherin, increasing membrane permeability and triggering downstream β-catenin signalling, which is a key pathway in CRC development^15,16^.

The importance of microorganisms in the contribution to cancer development is well established, with ‘polymorphic microbiomes’ considered to be an emerging cancer hallmark^17^. Improving our understanding of the role that bacteria play in CRC could allow the development of novel screening and treatment techniques and inform better preventative strategies. Bacterial biomarkers present in patient urine samples have shown promise in discriminating between benign colorectal adenomas and malignant carcinomas^18^, and antibiotic treatment of *F. nucleatum*-positive tumours has been shown to reduce tumour growth in murine models^19^. Furthermore, the administration of probiotic species such as *Escherichia coli* Nissle 1917 has been demonstrated to effectively treat inflammatory bowel disease^20^, inhibit the colonisation of intestinal pathogens^21^, and trigger apoptosis in the colorectal adenocarcinoma cell line HT-29 *in vitro*^22^.

There is clear potential for probiotic therapy in CRC treatment. However, it is currently unclear how bacterial involvement with CRC unfolds over time, with conflicting reports on the presence of specific pathogens in early and late-stage disease. The ‘alpha-bug’ model^23^, proposed by Sears and Pardoll, argues that specific species such as ETBF directly initiate CRC and remodel the microenvironment and composition of the microbiota to do so. In contrast, the ‘driver-passenger’ model^24^ proposes that initial driver bacteria promote the development of cancer and are then outcompeted by passenger bacteria which are better suited to the tumour microenvironment. This model is supported by the isolation of distinct populations of bacteria from colonic tissue at different stages of the adenoma carcinoma sequence^25^. The interaction with epithelial cells is central to the efficacy of bacteria to modify the growth and behaviour of tumour cells. It is therefore surprising that the question remains as to whether it is the tumour microenvironment or changes in the tumours cells themselves that promote association of specific species.

In vitro models allow the direct investigation of bacterial contact with colonic tumour cells. However, as many of these bacteria are strict anaerobes, complex co-culture systems are often employed to study these interactions, which are not suitable for assay development for use in basic research settings. Furthermore, there is a paucity of research investigating microbial interactions with benign tumour cells. To the authors’ knowledge, this is the first study to investigate the interaction of these CRC-associated species with benign colorectal tumour cells in vitro, which is necessary to identify which species are capable of driving progression to malignancy and metastasis. In the present study, bacterial interactions with colorectal adenoma and adenocarcinoma-derived cells were quantified in vitro by comparing the attachment, invasion and intracellular persistence of bacteria within the benign adenoma cell line S/RG/C2 and malignant carcinoma cell line HCT116. Once a model for infecting tumour cell monolayers had been established, the effects of this coculture on hallmark tumour cell behaviours, namely cell yield and migration, were investigated. The purpose is to study the interaction and invasion of bacterial species to support the future development of simple prognostic assays that could be used to identify individuals at high risk of developing CRC.

## Materials and methods

### Cell culture

The benign colonic adenoma cell line S/RG/C2^26^ and the malignant colonic adenocarcinoma cell line HCT116^27^ were cultured in high glucose Dulbecco’s modified eagle medium (DMEM; Sigma, Germany) at 37 °C and 5% CO_2_. Media for S/RG/C2 cells was supplemented with 20% FBS (Fisher Scientific, USA), 2% L-glutamine (Sigma), 0.2 U/ml human insulin (Sigma), 1 μg/ml hydrocortisone 21-hemisuccinate (Sigma) and 200 U/ml Penicillin–Streptomycin (Fisher Scientific). HCT116 media was supplemented with 10% FBS, 1% L-glutamine and 200 U/ml Penicillin–Streptomycin. HCT116 cells were purchased from ATCC (CCL-247). The S/RG/C2 cell line was originally derived at the University of Bristol. For gentamicin protection assays, S/RG/C2 and HCT116 cells were seeded into 96-well tissue cultures plates at a density of 1.6 × 10^5^ cells/cm^2^ and 4 × 10^4^ cells/cm^2^, respectively, and allowed to form confluent monolayers. The molecular characteristics of the cell lines used are summarised in Table 1.

**Table 1:** Selected molecular characteristics of the colorectal tumour cell lines S/RG/C2 and HCT116.

Cell line	Derivation	Origin	KRAS	BRAF	PTEN	TP53	PIK3CA
S/RG/C2	Colorectal villous adenoma	Adult male	wt	wt	wt	Arg^282^Trp	wt
HCT116	Colorectal adenocarcinoma	Adult male	G13D	wt	wt	wt	H1047R

### Bacterial culture

Enterotoxigenic *B. fragilis* (ATCC 43858), *E. coli* Nissle 1917 (NCIMB 775), *E. faecalis* (ATCC 19433) and *F. nucleatum* (ATCC 25586) were cultured on brain heart infusion (BHI) agar plates (Oxoid, Ireland) supplemented with 10% defibrinated horse blood (TCS Biosciences, UK) under anaerobic conditions (80% Nitrogen, 10% CO2, 10% Hydrogen at 80% humidity, 35 °C) in an A95 anaerobic workstation (Don Whitley Scientific Ltd. UK). Overnight cultures were prepared in BHI broth (Oxoid).

### Bacterial enumeration

Colony forming units (cfu) were determined by performing serial dilutions of bacteria in BHI broth followed by plating onto BHI agar plates supplemented with 10% defibrinated horse blood using the Miles and Misra method^28^. Bacteria were then incubated under anaerobic conditions for 24 h (facultative anaerobes) or 48 h (strict anaerobes) before colonies were quantified.

### Gentamicin protection assays

Tumour cells were seeded into 96-well plates and grown to confluency. Overnight cultures of bacteria were grown anaerobically, pelleted by centrifugation at 10,000 xG and resuspended in serum-free DMEM. Bacteria were then added to tumour cells at a multiplicity of infection (MOI) of 1000:1 and incubated at 37 °C and 5% CO2 for 4 h. Following infection, cell monolayers were washed three times with PBS (Fisher Scientific).

For bacterial attachment, monolayers were lysed immediately after washing with a solution of 1% w/v Saponin (Sigma) in serum-free DMEM. Bacteria were then serially diluted and enumerated on blood agar plates. For internalisation, monolayers were washed in PBS before being treated with 300 µg/ml gentamicin (Sigma) and 200 µg/ml metronidazole (Sigma) in serum-free DMEM to eliminate extracellular bacteria. Monolayers were washed a further three times with PBS, followed by tumour cell lysis in Saponin and bacterial enumeration as described above. The survival of bacteria within tumour cell monolayers was assessed by adding fresh media after antibiotic treatment. Penicillin–streptomycin was added to this media to prevent the outgrowth of bacteria released from the monolayer. Tumour cell lysis and bacterial enumeration was then performed at 24-h intervals up to 96 h post-infection.

### Tumour cell yield

S/RG/C2 and HCT116 cells were seeded into 12-well plates at a seeding density of 4 × 10^4^ cells/cm^2^ or 2 × 10^4^ cells/cm^2^, respectively. After 72 h tumour cells were treated with bacteria as described above. Following antibiotic treatment to eliminate extracellular bacteria, tumour cells were washed in PBS and fresh media was added. Cells were incubated for 96 h before being counted using a haemocytometer. Previous work in the Williams laboratory has demonstrated that in colorectal cell line cultures the vast majority > 80% of cells which detach from the culture surface and float in the supernatant are apoptotic. Conversely, attached cell numbers demonstrate small proportions of apoptotic cells (0 to 2%)^29^. Therefore, counting of floating cells in culture was used to approximate apoptotic cell numbers^29^.

### Migration and invasion assays

#### Wound healing

Tumour cells were seeded into 6-well plates and grown to confluence. Cells were then infected with bacteria at an MOI of 10:1 as described above. Following antibiotic treatment, a sterile P200 pipette tip was used to create a wound in the tumour cell monolayer. DMEM supplemented with 1% FBS was then added to inhibit cellular proliferation. Monolayers were imaged at 0 and 24 h, after washing in PBS to remove debris. The wound surface area was calculated using the MRI Wound Healing Tool for ImageJ.

#### Transwell migration and invasion

Tumour cells were seeded into 8 μm hanging cell culture inserts (Corning, USA) which were coated on the basal surface with 10 μg/ml VitroCol human collagen type 1 (Advanced Biomatrix, USA) for migration experiments, or were coated with Matrigel (Corning) for invasion experiments. Tumour cells were treated with bacteria, followed by antibiotics to eliminate extracellular bacteria. S/RG/C2 and HCT116 were resuspended in Ca^2+^-free DMEM (Fisher Scientific) and seeded into the apical compartment of the transwell at seeding densities of 4 × 10^4^ cells/cm^2^ and 2 × 10^4^ cells/cm^2^, respectively. Ca^2+^-free medium supplemented with 5% FBS was added to the basal chamber to act as a chemoattractant. After 24 h, transwells were washed and cells were fixed in ice cold methanol. Remaining cells on the apical surface were removed with a sterile cotton swab. Cells on the basal surface were considered migratory/invasive and were stained with 1% w/v crystal violet solution and counted in 10 fields of view at 200 × magnification.

### Microscopy

#### Phase contrast microscopy

Wound scratch images were captured using a Nikon TE300 inverted microscope (Meyer Instruments, USA).

#### Scanning electron microscopy

The interaction of bacteria with the surface of HCT116 cells was investigated using a FEI Quanta 650 field emission scanning electron microscope (Fisher Scientific, USA). Tumour cells were seeded at a low density in 24-well plates containing 10 mm coverslips and infected with bacteria at an MOI of 1000:1. Following infection, samples were fixed in 4% v/v glutaraldehyde (Sigma) in PBS. Samples were then dehydrated in increasing concentrations of ethanol, followed by hexamethyldisilazane (Fisher Scientific), before being gold sputter coated and mounted for imaging.

### Biofilm formation assays

Overnight cultures of bacteria were diluted 1:100 in BHI broth, or BHI broth supplemented with porcine mucin (Sigma). One hundred microliters of culture was then pipetted into a 96-well plate well and incubated under anaerobic conditions for 48 h. Liquid cultures were discarded, and biofilms adhered to the well surface were washed twice with sterile water and stained with 0.1% w/v crystal violet solution (Sigma). After staining, plates were washed a further three times before crystal violet solubilisation in 33% acetic acid (Sigma). An aliquot of this solution was added to a new 96-well plate and absorbance at 500 nm was read using an Infinite 200 microplate reader (Tecan, Switzerland).

### Data analysis and availability

All data analysis was performed using Graphpad Prism v8.2.0 for Windows (Graphpad Software) using two-tailed T tests to determine statistical significance between groups. All data are displayed as mean ± SEM with P ≤ 0.05 being considered significant. Image analysis was performed using FIJI (FIJI is Just ImageJ) for Windows (NIH). All data presented in this study is available fromhttps://doi.org/10.6084/m9.figshare.21310797

## Results

### Four species of gut microbes adhere more readily to cells derived from a benign adenoma than to a malignant carcinoma cell line

**Figure 1:**
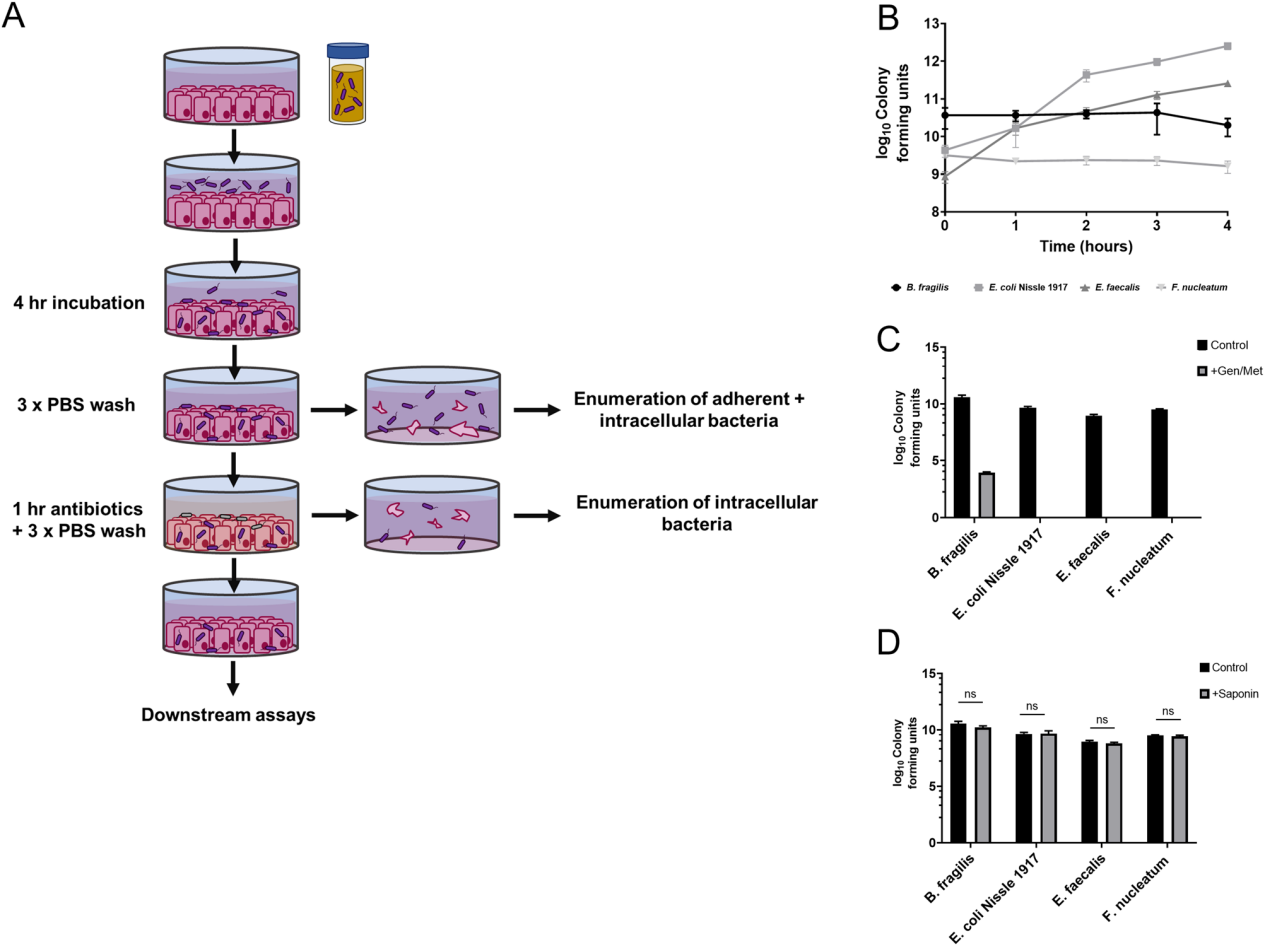
Optimisation of a simple model for co-culturing gut bacteria and colorectal tumour cells. (**A**) Schematic diagram of the model used, indicating points at which data on bacterial interactions with cells were collected or cells were used for downstream experiments. (**B**) Survival of chosen bacterial species under standard cell culture conditions (37 °C and 5% CO_2_) during the treatment period. (**C**) Administration of 300 μg/ml gentamicin and 200 μg/ml metronidazole effectively eliminated bacteria under the experimental conditions. (**D**) A 1% w/v saponin solution in DMEM cell culture media was used to lyse tumour cells and had no significant effect on bacterial viability.

In order to investigate the interactions of gut microbes with colorectal tumour cells and the subsequent downstream effects a simple co-culture model was devised (Fig. 1A). Tumour cells were grown to confluent monolayers, before a 4-h co-incubation under standard aerobic cell culture conditions. Tumour monolayers were then lysed after washing or antibiotic treatment to determine bacterial attachment and invasion, respectively, or used for further assays to determine tumour cell behaviour. All of the four bacterial species used throughout this study showed no reduction in viability during the 4-h co-culture, despite *B. fragilis* and *F. nucleatum* being considered to be strict anaerobes (Fig. 1B). To eliminate extracellular bacteria for invasion quantification, and to produce infected tumour cells for downstream assays, an antibiotic cocktail of 300 μg/ml gentamicin and 200 μg/ml metronidazole in serum-free DMEM was used. These concentrations were confirmed to eliminate viable bacteria in our samples, with the exception of *B. fragilis* cultures where a > 5 log reduction was observed (Fig. 1C). Finally, the saponin solution used for tumour cell lysis was also confirmed to have no negative impact on bacterial viability (Fig. 1D). Taken together, these results confirmed the suitability of our co-culture model for investigating the interactions of these species within colon tumour cell lines.

**Figure 2:**
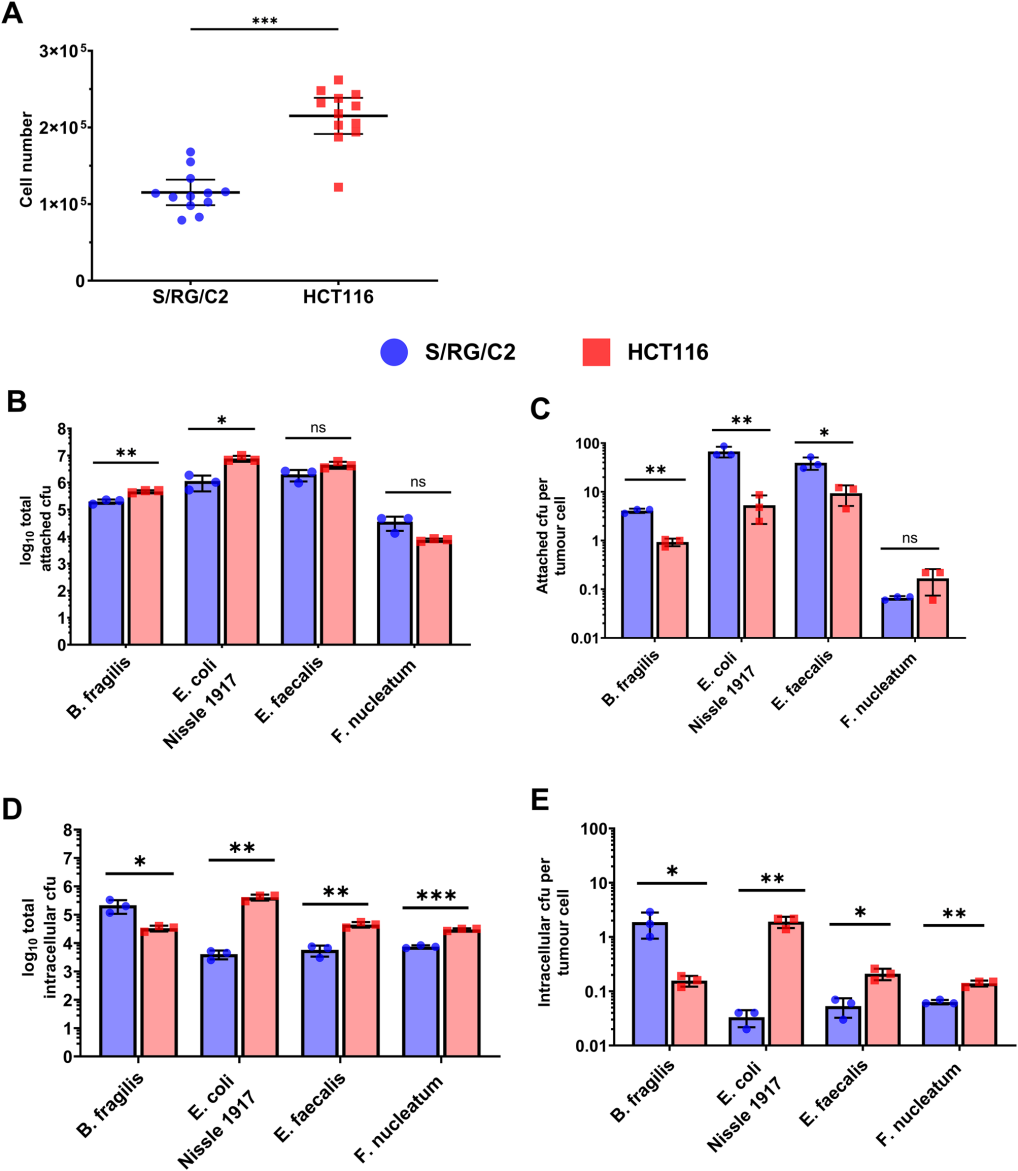
(**A**) Comparison of the total cell numbers in colorectal tumour cell monolayers cultured in 96-well plates after cells were cultured to confluency for 1 week. Bacterial attachment to tumour cell monolayers (**B** and **C**) and bacterial invasion of tumour cell monolayers (**D** and **E**) were quantified and expressed as both total bacteria per monolayer and average bacteria per tumour cells. * = *p* < 0.05, ** = *p* < 0.01, *** = *p* < 0.001.

The S/RG/C2 cell line was initially isolated from a benign sporadic colorectal adenoma and are slower growing cells than the HCT116 cells, which form uniform monolayers comprised of greater cell numbers (Fig. 2A). All four species of bacteria tested readily adhered to the surface of both S/RG/C2 and HCT116 monolayers after a 4-h co-culture period (Fig. 2B and C). The total number of bacteria present at the tumour cell surface ranged from 1 × 10^4^ to 1 × 10^7^ for all bacteria species tested. Interestingly, after normalising to cell number, all species, with the exception of *F. nucleatum,* demonstrated significantly higher adherence to benign S/RG/C2 monolayers than malignant HCT116 cells. Drawing comparisons in adhesive capabilities between species is difficult, as replication of the facultative anaerobes *E. coli* Nissle and *E. faecalis* was observed during the optimisation of the assay parameters (Fig. 1B). This may explain why the recovered colony forming units (cfu) for these species was far greater than that of *B. fragilis* and *F. nucleatum*.

### Gut bacteria show higher levels of intracellular invasion into malignant cells than benign cells

In contrast to adherence to the cell surface, bacterial invasion (Fig. 2D and E) of colorectal tumour cell monolayers was higher in HCT116 cells than S/RG/C2 cells, with the exception of *B. fragilis*, where an average of 1.88 bacterial cells were detected in the adenoma line, compared with 0.15 cells per carcinoma cell. The largest difference in invasive capabilities between S/RG/C2 and HCT116 monolayers was observed for *E. coli* Nissle, with an average of 0.03 bacteria per cell in S/RG/C2 co-cultures and 1.91 bacteria per cell in HCT116 co-cultures (Fig. 2E). *E. faecalis* and *F. nucleatum* invasion of colorectal cell lines was also higher in HCT116 cells, with an average of 0.21 and 0.14 cells per carcinoma cells, compared with 0.05 and 0.06 cells per adenoma cell respectively.

**Figure 3:**
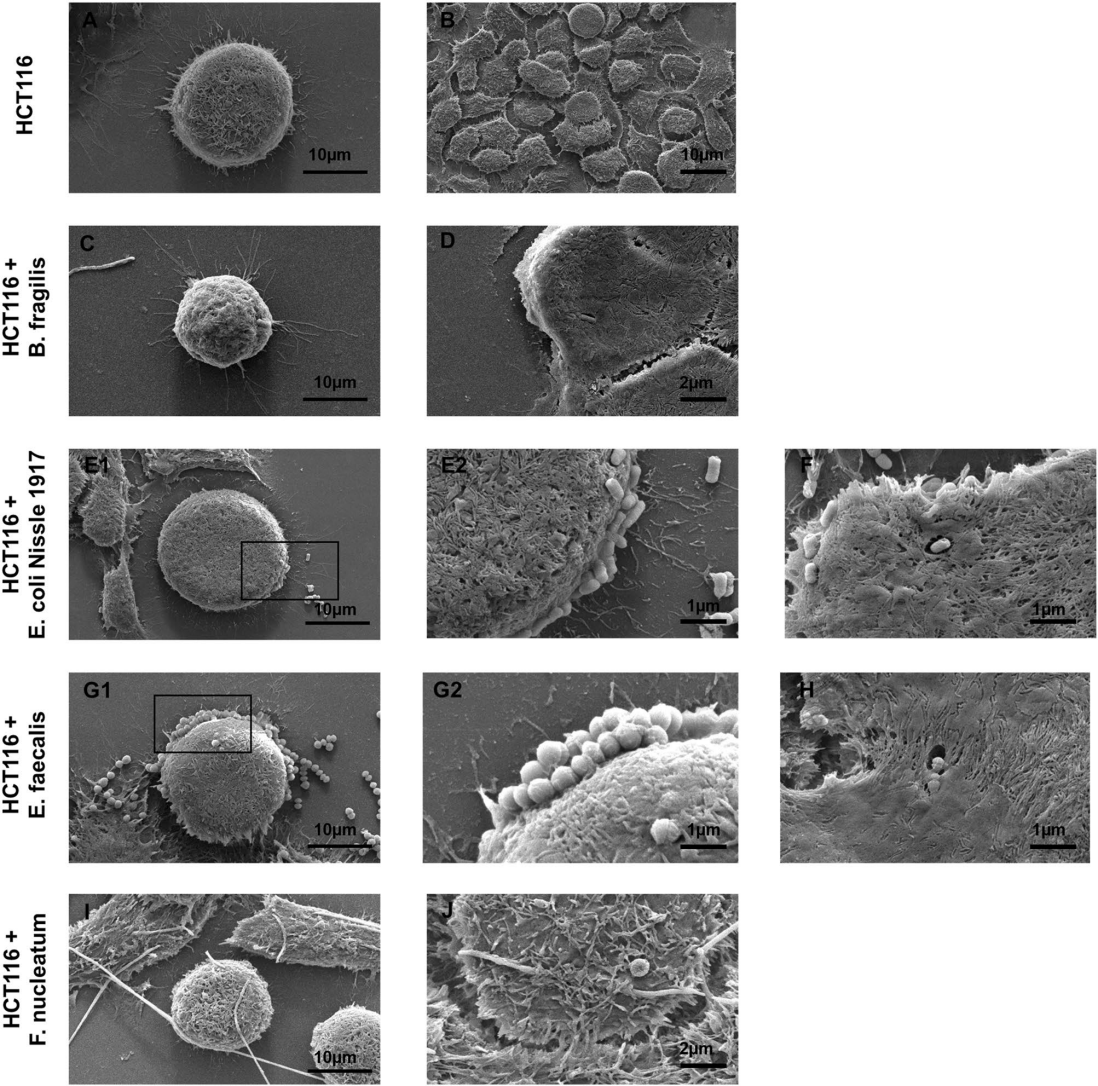
Scanning electron micrographs of HCT116 tumour cells after treatment with selected gut bacteria. (**A**) Morphology of an individual HCT116 cell. (**B**) HCT116 monolayer. *B. fragilis* was observed at the tumour cell surface (**C**) and within ‘pit-like’ pores on the tumour cell surface (**D**). Both *E. coli* Nissle 1917 (**E**) and *E. faecalis* (**G**) were observed at the tumour cell surface in co-aggregations, in addition to being observed within pore structures (**F** and **H**). Black boxes indicative regions magnified in (**E2**) and (**G2**). *F. nucleatum* was observed as long (> 10 μm) cells, which made contact with several tumour cells (**I** and **J**). Scale bares represent 10 μm).

To visualise bacterial adherence to colorectal tumour cells, HCT116 cells were grown at low density to better visualise single cells, and co-cultured with bacteria as described above. In isolation, HCT116 cells display a rounded morphology (Fig. 3A), which becomes more polygonal at regions of higher density (Fig. 3B). All four species of bacteria tested were imaged in association with the tumour cell surface. With the exception of *F. nucleatum*, all species were also observed in pore-like structures on the tumour cell surface (Fig. 3D,F,H). The facultative anaerobes *E. coli* Nissle and *E. faecalis* were found to co-aggregate at the cell surface in large numbers (Fig. 3E + G), this is in contrast to *B. fragilis* which was often observed in single cell–cell adhesions (Fig. 3C). The morphology of *F. nucleatum* cells appeared variable, with cell length of between 5 and 30 μm observed during these experiments (Fig. 3I,J). This suggests that these bacteria may alter their morphology and become shortened during adherence to mammalian cells, and that this morphology may determine their interaction with the gut epithelium, although this requires further investigation.

### Gut bacteria persist within tumour cell monolayers in a cell line-dependent manner

**Figure 4:**
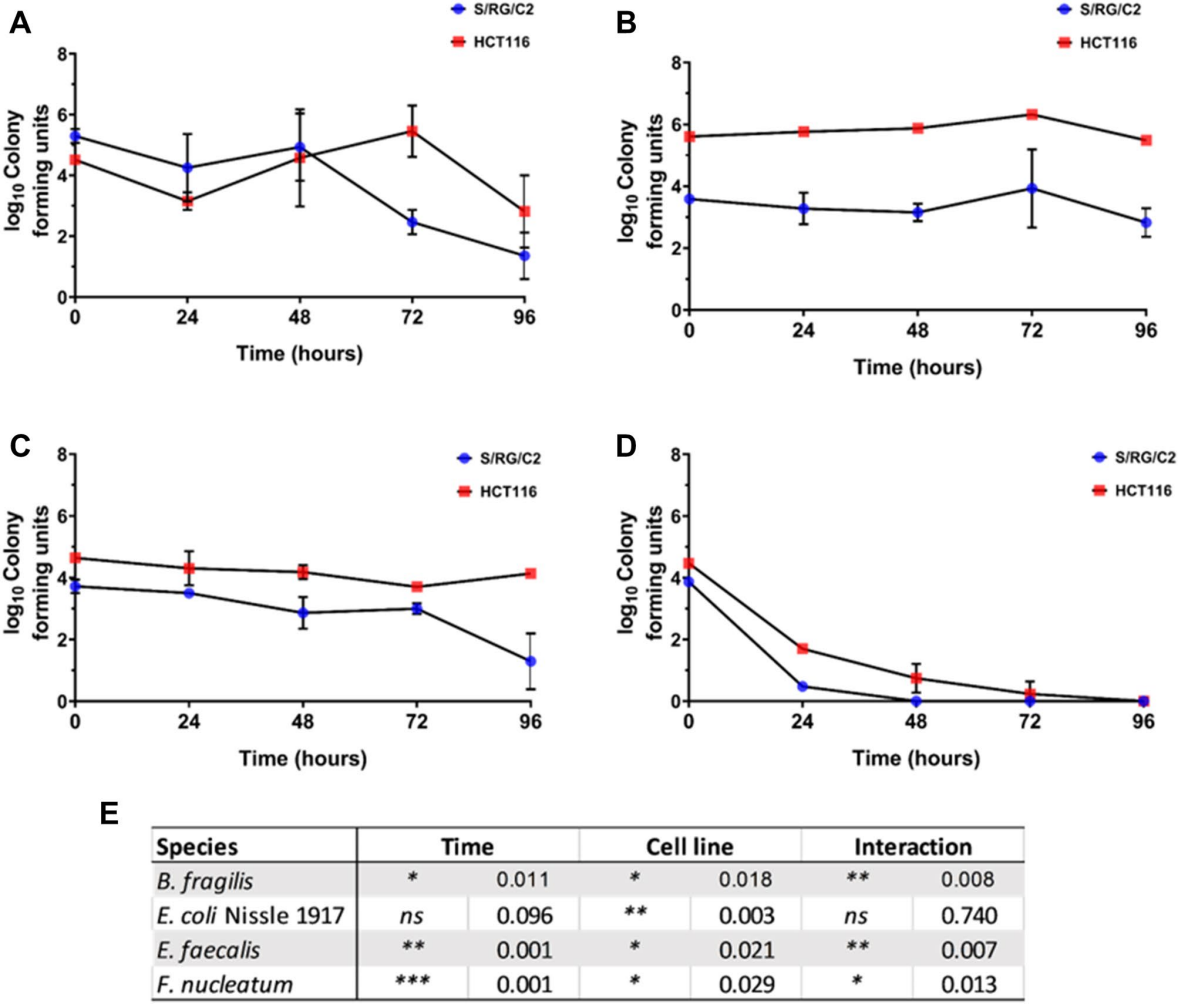
Persistence *B. fragilis* (**A**), *E. coli* Nissle 1917 (**B**), *E. faecalis* (**C**) and *F. nucleatum* (**D**) within colorectal tumour cell monolayers under standard cell culture conditions. Table below indicates the results of a Two-way ANOVA examining the relationship between bacterial survival, cell line and time. * = *p* < 0.05, ** = *p* < 0.01, *** = *p* < 0.001.

The ability of bacteria to persist within the tumour cell monolayers was investigated by continuing tumour cell-bacteria co-cultures after eradication of extracellular bacteria under standard aerobic tissue culture conditions. All species were recovered from both S/RG/C2 and HCT116 monolayers after 48 h of continued culture (Fig. 4). *F. nucleatum* was not isolated from tumour cells after this time, but all other species were found to persist within tumour monolayers for the entire 96-h assay period. A two-way ANOVA was performed to determine whether survival of bacteria with S/RG/C2 or HCT116 monolayers was statistically different, accounting for differences in the initial number of intracellular bacteria (Fig. 4E). The survival of *B. fragilis*, *E. faecalis* and *F. nucleatum* was significantly higher in HCT116 cells than in S/RG/C2 cells, with no statistically significant difference in the survival of intracellular *E. coli* Nissle was recorded between the two cell lines tested.

### Mucin alters bacterial virulence in vitro

**Figure 5:**
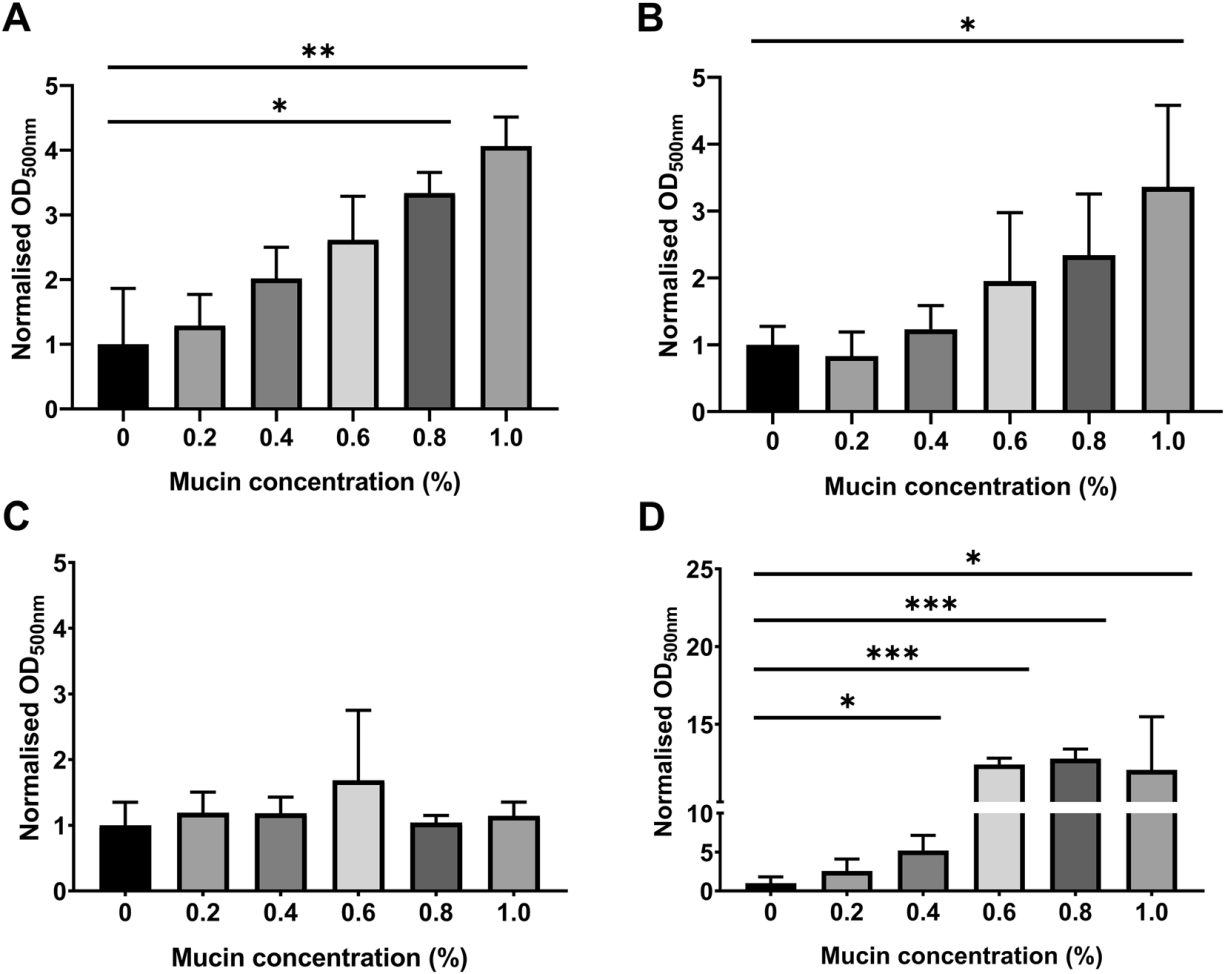
Crystal violet biofilm assay to determine the effects of mucin on the biofilm forming capabilities of *B. fragilis* (**A**), *E. coli* Nissle 1917 (**B**), *E. faecalis* (**C**) and *F. nucleatum* (**D**) under anaerobic conditions. * = *p* < 0.05, ** = *p* < 0.01, *** = *p* < 0.001.

Clear differences in bacterial association were observed between S/RG/C2 and HCT116 monolayers. The benign S/RG/C2 cell line retains several characteristics of normal colonic epithelium including microvilli and mucin secretion^30^. To determine whether gastrointestinal mucin causes a phenotypic change in gut bacteria which may alter their virulence, the four species tested were cultured with or without porcine intestinal mucus and their biofilm forming capabilities were quantified using the crystal violet biofilm assay. Three species, *B. fragilis*, *E. coli* Nissle 1917 and *F. nucleatum* displayed a dose-dependent increase in biofilm formation when cultured in the presence of mucin (Fig. 5). For these species, the increase in biofilm formation was statistically significant at mucin concentrations of 1.0%. *F. nucleatum* biofilm formation also significantly increased at lower mucin concentrations, suggesting this species may be more sensitive to mucin factors. Mucin had no effect on the biofilm forming capabilities of *E. faecalis*, which despite showing the highest absorbance readings across all species showed a high degree of variability in its biofilm formation.

### Co-culture with gut microbes increases tumour cell yield in both benign and malignant cell lines

**Figure 6:**
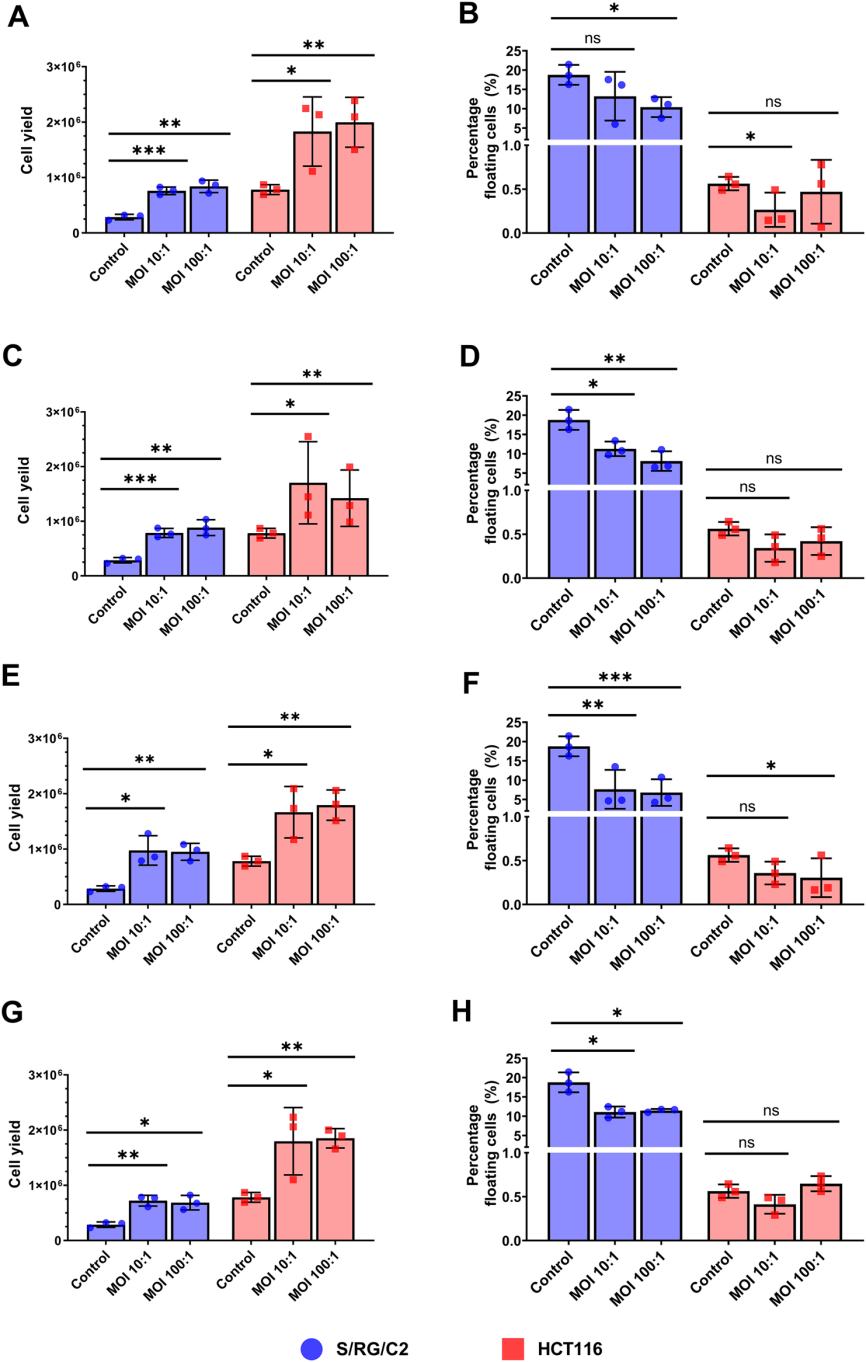
Viable and floating cell counts of HCT116 and S/RG/C2 cells after 4 h treatment with *B. fragilis* (**A** + **B**), *E. coli* Nissle 1917 (**C** + **D**), *E. faecalis* (**E** + **F**) and *F. nucleatum* (**G** + **H**). . * = *p* < 0.05, ** = *p* < 0.01, *** = *p* < 0.001.

After the novel finding that the four bacterial species invaded and persisted within both S/RG/C2 and HCT116 tumour cells, sub-confluent cultures were infected at MOIs of 10:1 and 100:1, and the resulting effect on tumour cell yield was quantified 96 h post-treatment. As the ability of the chosen microorganisms to interact with colon cell monolayer was now established, lower MOIs were selected for long term culture experiments investigating effects on tumour cells yield to better mimic in vivo conditions, where the majority of bacterial cells are not in contact with the luminal surface^31^. Bacterial co-culture with all four species tested significantly increased tumour cell yield in both S/RG/C2 and HCT116 cells by a factor of at least 1.5 (Fig. 6A,C,E,G). Across all species and MOIs, the average cell yield in infected S/RG/C2 cells was 8.3 × 10^5^ cells/well, a value greater than the cell yield observed in untreated HCT116 cells. This suggests that under the conditions of this experiment, bacterial invasion can promote a growth phenotype in benign tumour cells similar to that of malignant cancer cells.

Colon tumour cells grown in vitro detach from the culture surface when undergoing apoptosis, and therefore cells floating in the supernatant can be quantified as a direct measure of apoptosis^29,32^. Counts of floating apoptotic cells were performed in parallel to cell yield counts. In treated S/RG/C2 cultures, apoptosis was significantly reduced by all four bacterial species tested (Fig. 6B,D,F,H)). Reductions in the rate of apoptosis were also observed in HCT116 co-cultures, however; this was only statistically significant in cultures treated with *B. fragilis* at an MOI of 10:1 and E. faecalis at an MOI of 100:1.

### Cancer-associated* F. nucleatum* stimulates both migration and invasion of malignant tumour cells in vitro but not benign tumour-derived cells

**Figure 7:**
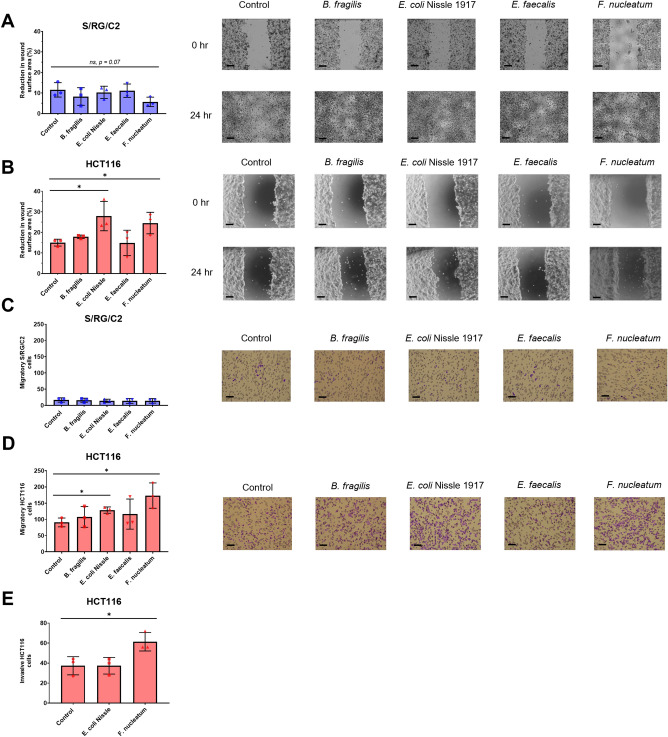
The effects of bacterial treatment on colorectal tumour cell migration and invasion. (**A**) S/RG/C2 wound scratch assay. (**B**) HCT116 wound scratch assay. (**C**) S/RG/C2 transwell filter migration. (**D**) HCT116 transwell filter migration As significant effects on migration were only observed for HCT116 cells treated with *E. coli* Nissle 1917 and *F. nucleatum*, these conditions were used in a transwell filter invasion assay (**E**). Wound scratch and transwell filter migration images were captured at 10 × and 20 × objective magnification, respectively. Wound scratch scale bars represent 50 µm, transwell filter scale bars represent 25 µm. * = *p* < 0.05.

Along with tumour growth, cancer cell migration and invasion are key determinants of disease progression to metastasis. To determine whether these bacteria were able to influence tumour cell motility, we performed wound healing assays on tumour cell monolayers 24 h post-bacterial treatment. After monolayers reached confluency, a sterile P200 pipette tip was used to create a wound, which was photographed at 0- and 24-h post-creation. In S/RG/C2 cell monolayers, significant debris is observed during wound healing assays, consistent with the high rates of apoptosis typical in this cell line in Fig. 6. For S/RG/C2 cells. No statistically significant effect on wound healing was observed in S/RG/C2 co-cultures (Fig. 7A). However, infection of HCT116 monolayers with both *E. coli* Nissle and *F. nucleatum* significantly increased wound healing (Fig. 7B). This result was then confirmed using a transwell filter migration assay, whereby cells able to migrate through a collagen-coated cell culture inserts are counted Similar to the wound healing assay, there were no significant effects of bacterial co-culture on S/RG/C2 migration. However, migration of the malignant HCT116 cell line was significantly increased by *E. coli* Nissle and *F. nucleatum* infection (Fig. 7C,D). Invasion by tumour cells into the surrounding tissue requires not only motile characteristics but also the degradation of surrounding extracellular matrix. To test whether the increase in HCT116 motility caused by *E. coli* Nissle and *F. nucleatum* infection was also reflected in invasion behaviour, Matrigel®-coated transwell filter membranes were used. To pass through Matrigel®-coated inserts, cells must be capable of remodelling extracellular matrix through the production of proteinases and other enzymes, in addition to physically migrating*. nucleatum* was able to significantly increase HCT116 invasion, with an average of 61 invasive cells counted per field of view, compared to 37 invasive cell counted in non-treated controls (Fig. 7E).

## Discussion

Colonic bacteria have been implicated in the development and progression of CRC. Certain species are found at greater proximity to the mucosal surface^33^, isolated in greater abundance from CRC patients than healthy controls^11^, display higher densities at privileged sites^34^ and can be isolated directly from tumour tissue^25^. Whether this increased association between colonic microorganisms and colonic tumours is as a result of alterations to the mucosa caused by tumour formation, or whether these bacterial species actively colonise the gut at different stages of the adenoma-carcinoma sequence and promote this association remains unclear. In this paper, we demonstrate that anaerobic bacteria commonly associated with CRC remain viable under the conditions of the downstream assays performed. Furthermore, after these bacteria invade tumour cells, they are able to remain viable within the intracellular space for several days. This co-culture model then facilitated the investigation of the interactions between the bacteria and tumour cells, and the subsequent effects on both benign and malignant tumour cell behaviour.

The ability to adhere to and invade the mucosa is a key virulence factor which impacts the contribution of microorganisms to CRC^35,36^. Distinct microbiota populations have been associated with different stages of CRC development^11^. The Driver-Passenger model proposes that CRC-driving species are transiently associated with specific stages of tumorigenesis, as they are gradually replaced by species better adapted to the changing tumour microenvironment^24^. During the adenoma-carcinoma sequence changes to the tumour microenvironment and architecture including tumour size, surface area, expression of surface proteins, mucus secretion, cellular metabolism and tumour vasculature, all of which are likely to impact interactions with gut microorganisms^37–40^. This study investigated the interactions of three CRC-associated microorganisms, and one probiotic species, with benign S/RG/C2 and malignant HCT116 colorectal tumour cells. The S/RG/C2 cell line was initially isolated from a sporadic tubular adenoma^26^, and interestingly displays a morphology distinct from that of established adenocarcinoma cells in vitro.

When comparing bacterial adhesion between S/RG/C2 and HCT116 monolayers, significantly higher bacterial attachment to S/RG/C2 cells was observed, despite considerable numbers of bacteria also adhering to HCT116 cells. Bacterial adhesion to epithelial cell surfaces is often attributed to their binding to specific cell surface proteins^41–43^. Both *B. fragilis* and *F. nucleatum* have previously been demonstrated to interact with the epithelial junction protein E-cadherin the expression of which is greater in S/RC/C2 cells than in adenocarcinoma cell lines^30,44^. This may account for the increased levels of attachment seen to S/RG/C2 monolayers, particularly as HCT116 monolayers exhibited a greater cell number per monolayer. Furthermore, the potentially larger surface area provided by the three-dimensional S/RG/C2 culture may provide additional binding sites for bacteria. Finally, unlike HCT116 cells, S/RG/C2 cells growing in vitro produce exogenous mucin^26^, which is a key substrate for bacterial attachment in vivo^45,46^, altering the transcription of numerous microbial genes regulating virulence and pathogenicity in addition impacting cell signalling pathways in cancer^47^. Therefore, S/RG/C2-derived mucus may also contribute to the discrepancy in bacterial associated between the two cells lines.

Attachment of the facultative anaerobes *E. coli* Nissle and *E. faecalis* was higher than the strict anaerobes *B. fragilis* and *F. nucleatum*, however, this is likely to be in part due to the proliferation of these species during the co-culture period. In addition, scanning electron microscopy indicated that both *E. coli* Nissle and *E. faecalis* cells co-aggregate with other cells of the same species at the tumour cell surface, meaning that the recorded attached cfu may also represent non-attached cells which were bound to other attached bacteria. Despite this, the proximity of these cells to the tumour cell surface suggests it is likely that they would still be capable of influencing tumour cell behaviour through the secretion of metabolites and signalling to adjacent bacterial cells.

In contrast to bacterial attachment, bacterial invasion was significantly higher in HCT116 cells when compared to S/RG/C2, with the exception of *B. fragilis*. This suggests that adenocarcinoma cells may be predisposed to bacterial invasion, which is surprising given previous research indicating that species such as *F. nucleatum* invade intestinal epithelial cells through interactions with E-cadherin^16^. There is clinical precedent for greater invasion of advanced cancer cells, as bacteraemia with specific gut microbes as the causative agents is linked to the subsequent development of CRC^35,48^, although the altered microenvironment and immune status of the patient will also play a significant role. Finally, our biofilms assays have demonstrated that even small amounts of mucin can alter the phenotype of these species, and therefore mucin produce by S/RG/C2 cells may influence the cell line’s interaction with bacteria^26^. In colonic lumen, the mucin layer serves as both a protective barrier against and a substrate for microorganisms, with numerous ecological niches providing a habitat for thousands of species^49^.

After confirming that all four species tested were able to successfully infect S/RG/C2 and HCT116 cells during a 4-h infection period, this study also aimed to determine the ability of these bacteria to survive within the tumour monolayer under standard cell culture conditions. Although the survival of each species varied, all species were isolated from both S/RG/C2 and HCT116 tumour monolayers for at least 48 h post-infection. It is important to note that non-viable intracellular bacteria would not be detected using the current method, however, neither gentamicin or metronidazole are thought to be active against intracellular bacteria, even when present at bactericidal concentrations in the cell culture media^50^., With the exception of *E. coli* Nissle, all other species demonstrated significantly higher survival in HCT116 monolayers when compared with those of S/RG/C2 cells . It has recently been established that tumour-associated bacteria, including *F. nucleatum*, are able to persist within tumour cells in vivo during tumour metastasis^19^, and to the author’s knowledge this represents the first demonstration of in vitro persistence within colorectal tumour cells, potentially allowing for investigation into the mechanisms which facilitate this persistence, and the subsequent effects on tumour cell behaviour.

Despite the differing levels of bacterial association between S/RG/C2 and HCT116 tumour cells, bacterial treatment with all species had similar effects on cell yield, with significant increases in both cell lines. This may suggest that relatively small numbers of bacteria penetrating the mucus barrier in CRC are sufficient to drive changes to the tumour phenotype. This is consistent with current opinions on bacterial drivers and ‘alpha-bugs’ in CRC, which are thought to be present at very low concentrations but have disproportionately large effects on the microenvironment^23,24^. The concordance between the increases in cell yield observed in S/RG/C2 and HCT116 cells suggests that there exists a conserved, contact-dependent mechanism through which bacteria are able to upregulate cell yield, warranting further investigation. Parallel counts of floating cells above tumour cultures revealed that bacteria are also able to reduce apoptosis within colon tumour cell populations. Apoptosis is a key regulator of normal gut homeostasis which is disrupted in CRC. In the healthy colon, epithelial cells are continually sloughed into the lumen^51^, a process which is reversed in CRC development with reduced apoptosis at the luminal surface and increased crypt apoptosis^52^. Our data demonstrates that bacterial interactions with colonic epithelial cells may reduce the apoptosis allowing for further tumour growth and accumulation of pro-oncogenic mutations.

The activation of invasion and metastasis is one of the key hallmarks of cancer, with metastasis responsible for an overwhelming majority of cancer-related deaths^53,54^. To determine the impact of the test bacterial species on tumour cell migration and invasion we employed simple wound scratch experiments coupled with both transwell filter migration and invasion assays. As expected, the S/RG/C2 cell line originally isolated from a benign adenoma displayed lesser migratory potential than the malignant HCT116 cell line in the transwell filter migration assay. Interestingly, differences between the two cell lines were not observed in wound healing assays, suggesting that it is in single cell migration where the key different in migratory potential lies. Normal epithelial cells require a degree of motility in order to repair wounds, which primarily occurs via sheet migration^55^. This is important to consider as tumour cells are known to migrate and metastasise using several phenotypes, however single cell migration is not a feature of normal epithelial cells^56^. Upon bacterial treatment, both *E. coli* Nissle 1917 and *F. nucleatum* caused an increase in migration in HCT116 cells, but not in S/RG/C2 cells. These results were confirmed in both of the methodologies used. Previous studies have demonstrated that S/RG/C2 cells can be stimulated to migrate^57^, suggesting that this bacterial interaction is sufficient to promote motility in pre-motile cells, but cannot confer a migratory phenotype in benign cells. *F. nucleatum* was the only species capable of promoting an increase in HCT116 invasion. When considering the Driver-Passenger model of bacterial contribution to CRC development, this would frame these species as drivers of late-state metastatic disease. A role for *F. nucleatum* in colorectal tumour motility through its interactions with E-cadherin has previously been suggested^16^. However, our results would indicate that an E-cadherin-independent mechanism may also exist, as no effect on the migration of S/RG/C2 was observed despite this cell line possessing higher E-cadherin expression than adenocarcinoma cell lines such as HT29^30,32^. *F. nucleatum* can be detected in both primary and CRC tumours and their distant metastases, and abrogation of *F. nucleatum* populations through antibiotic administration improve survival^19^. Therefore, the results of the present study strengthen the argument for antibiotic adjuvant therapy in CRC cases with confirmed *F. nucleatum* presence.

In conclusion, our results demonstrate that CRC-associated microorganisms interacted differently with tumour cells isolated from benign and malignant tumours. Despite these differences, all species tested were able to induce significant increases in tumour cell yield. One species, *F. nucleatum* was found to increase both migration and invasion, but this effect was restricted to malignant HCT116 cells. This work contributes to the growing case for viewing the microbiome as a key regulator of CRC progression, with further research needed to unravel the complex relationship between CRC development and microbial association.

## Data Availability

The data that support the findings of this study are available in ‘FigShare’ at https://doi.org/10.6084/m9.figshare.21310797
